# Arginine for the Treatment of Mitochondrial Encephalopathy, Lactic Acidosis, and Stroke-Like Episodes: A Systematic Review

**DOI:** 10.7759/cureus.32709

**Published:** 2022-12-19

**Authors:** Jennifer M Argudo, Olga M Astudillo Moncayo, Walter Insuasti, Gabriela Garofalo, Alex S Aguirre, Sebastian Encalada, Jose Villamarin, Sebastian Oña, Maria Gabriela Tenemaza, Ahmed Eissa-Garcés, Sakina Matcheswalla, Juan Fernando Ortiz

**Affiliations:** 1 School of Medicine, Universidad de Cuenca, Cuenca, ECU; 2 Division of Research and Academic Affairs, Larkin Community Hospital, Miami, USA; 3 School of Medicine, Universidad Central del Ecuador, Quito, ECU; 4 School of Medicine, Universidad San Francisco de Quito, Quito, ECU; 5 School of Medicine, Universidad de las Americas, Quito, ECU; 6 Department of Neurology, Universidad San Francisco de Quito, Quito, ECU; 7 College of Medicine, Krishna Institute of Medical Sciences, Mumbai, IND; 8 Department of Neurology, Corewell Health, Michigan State University, Grand Rapids, USA

**Keywords:** mitochondrial encephalopathy, melas syndrome, l-arginine, mitochondrial disease, lactic acidosis, stroke, melas

## Abstract

Mitochondrial encephalopathy, lactic acidosis, and stroke-like episodes (MELAS) syndrome is a mitochondrial disease that lacks a definitive treatment. Lately, there has been an increased interest in the scientific community about the role of arginine in the short and long-term settings of the disease. We aim to conduct a systematic review of the clinical use of arginine in the management of MELAS and explore the role of arginine in the pathophysiology of the disease. We used PubMed advanced-strategy searches and only included full-text clinical trials on humans written in the English language. After applying the inclusion/exclusion criteria, four clinical trials were reviewed. We used the Meta-analysis of Observational Studies in Epidemiology (MOOSE) guidelines and Preferred Reporting Items for Systematic Reviews and Meta-Analyses (PRISMA) protocol for this systematic review. We used the Cochrane Collaboration risk-of-bias tool to assess the bias encountered in each study. Overall, IV arginine seems to be effective in improving symptoms during acute attacks of MELAS, while oral arginine supplementation increases endothelial function, preventing further stroke-like episodes.

## Introduction and background

Mitochondrial encephalopathy, lactic acidosis, and stroke-like episodes (MELAS) syndrome is a frequent maternally inherited mitochondrial disorder with a broad spectrum of manifestations due to multiorgan involvement succeeding insufficient adenosine 5′-triphosphate (ATP) production [1]. The 3243 A>G point mutation in the mitochondrial transfer RNA (tRNA) has been associated with MELAS, presenting an impaired taurine modification at the first base of the tRNA anticodon against the wobble base pair [1,2]. This mutation has been found in other neurologic and non-neurologic diseases [1]. Although the 3243 A>G point mutation is a common mutation found in MELAS patients, other mutations may be present as well, such as the case of mutation hotspots in the *MTND* gene [3]. Due to the nature of the disease, the brain and muscles are always affected by mitochondrial dysfunction, and those who are diagnosed with MELAS present with more than one clinical feature [4]. Common clinical manifestations of MELAS include nausea and vomiting, seizures, headaches, dementia, peripheral neuropathy, stroke-like episodes, learning disability, short stature, and lactic acidosis [1]. Cardiac involvement is also a very common feature as it affects >55% of patients with the 3243 A>G mutation. Left ventricular hypertrophy is the most common cardiac presentation of MELAS, frequently evolving into dilated cardiomyopathy. Atrioventricular blocks and arrhythmias may also occur [1,5,6]. Diagnostic criteria for MELAS were first proposed in 1940 by Pavlakiest et al., and include the onset of symptoms at the age of three to 11 years, normal early development, short stature, lactic acidosis, seizure, alternating hemiparesis, hemianopia, parieto-occipital lucencies, and ragged red fibers [7]. However, in 2012, a prospective cohort study conducted by the MELAS study committee in Japan established the categories for diagnostics, as shown in Table 1 [2].

**Table 1:** Diagnostic criteria for MELAS according to the MELAS study committee in Japan Definitive MELAS: defined by two items in category A and two items in category B. Suspicion of MELAS: one item in category A and two items in category B. MELAS: mitochondrial encephalopathy, lactic acidosis, and stroke-like episodes.

Category A. Clinical findings of stroke-like episodes
Headache
Seizures
Hemiplegia
Cortical blindness or hemianopsia
Acute focal lesion observed in brain imaging
Category B. Evidence of mitochondrial dysfunction
High lactate levels in plasma and/or cerebral spinal fluid or deficiency, mitochondrial-related enzyme activities
Mitochondrial abnormalities in muscle biopsy
Definitive gene mutation related to MELAS

Other tools such as the Japanese Mitochondrial Disease Rating Scale (JMDRS) and the Newcastle Mitochondrial Disease Adult Scale (NMDAS) are commonly used to evaluate the progression of illness in patients being treated for mitochondrial disease [7,8].

The treatment of MELAS is complex and mainly unknown. Numerous drugs have been investigated for the treatment of MELAS, such as the supplementation with glutamine and dichloroacetate. The latter is used cautiously, given its potential to cause toxic peripheral neuropathy in MELAS patients [9]. Table 1 shows the diagnostic criteria for MELAS [9].

Taurine with arginine supplementation has been shown to decrease the frequency of stroke-like episodes in one open-label study [10]. However, the effects of taurine have not been investigated alone [10].

Anticonvulsants are commonly prescribed in MELAS patients, given their use in preventing and treating status epilepticus as well as delaying brain atrophy. Nevertheless, anti-epileptic drugs that are less mitochondrial toxic should be preferred [11-13].

Lately, greater interest has appeared in the use of arginine in the treatment of MELAS. The objective of this study is to conduct a systematic review of the clinical trials of arginine in the treatment of MELAS. Additionally, we aim to investigate the pathophysiology of the disease and establish a rationale for the use of arginine in the treatment of MELAS.

## Review

Materials and methods

Protocol

We carried out a systematic review guided by the standards of the Preferred Reporting Items for Systematic Reviews and Meta-Analyses (PRISMA) [14].

Eligibility Criteria and Study Selection

Independent extraction of articles was performed by two authors to assess eligibility. The inclusion criteria included full-text clinical trials written in the English language and conducted on humans. We excluded case reports, observational studies, literature reviews, systematic reviews, and meta-analyses for the analysis of our paper. After screening the studies, we only included articles with the following characteristics: (1) patients: patients diagnosed with MELAS; (2) intervention: L-arginine; (3) comparator: control group; and (4) outcomes: death, disability, and functional outcome. Disagreements between reviewers were solved by consensus. Furthermore, we hand-searched the articles used to exclude duplicated results.

Database and Search Strategy

The search was applied to the PubMed (2000-present) and Google Scholar (2000-present) databases. We used an advanced search strategy to search all clinical trials with the following terms: L-arginine (title/abstract) and MELAS (title/abstract). The last search was conducted on April 12, 2022.

Data Extraction and Analysis

The following information from each article was collected: (1) methods including dose, duration, route of administration, number of participants, study design, and patient selection criteria; (2) main results, including the outcome measures and main limitations in each clinical trial; (3) lastly, the primary and secondary outcomes and main conclusions.

Bias Assessment

We used the Cochrane Collaboration risk-of-bias tool to assess the bias encountered in each study [15].

Results

**Figure 1:**
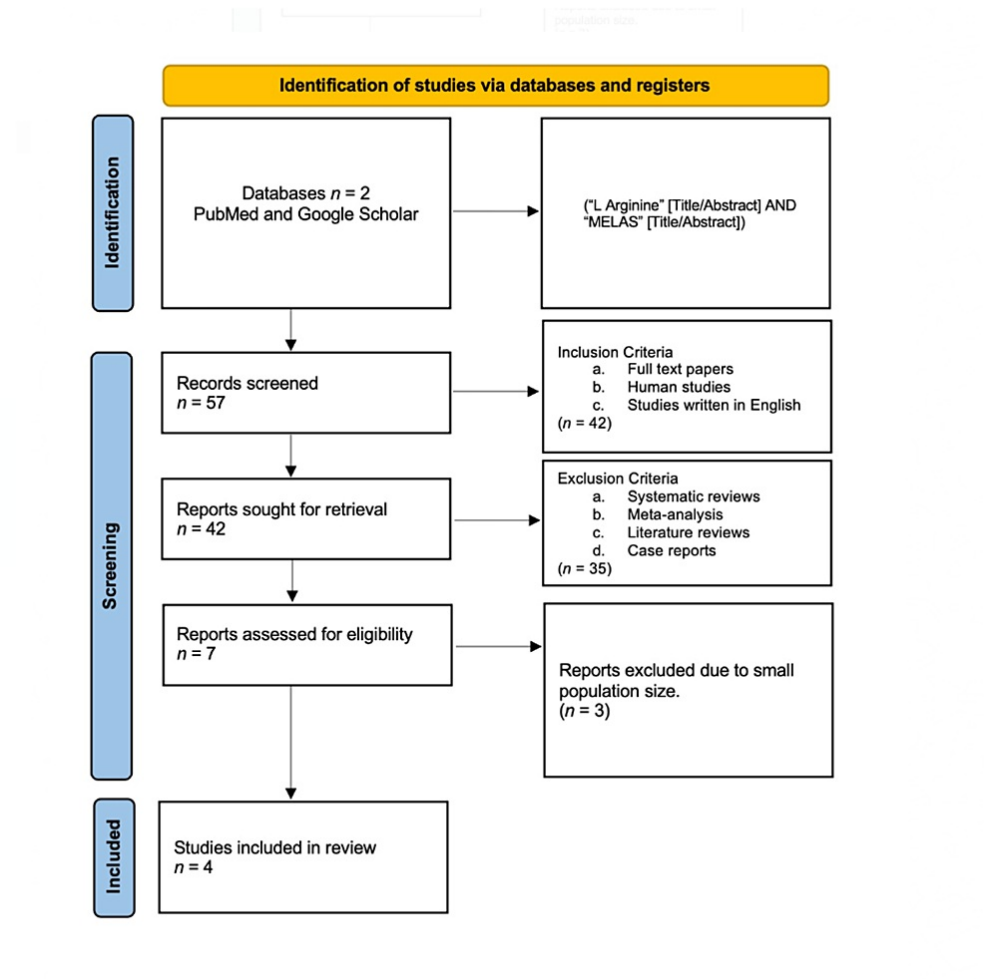
PRISMA flow chart showing the results of the research conducted PRISMA: Preferred Reporting Items for Systematic Reviews and Meta-Analyses.

We found six clinical trials and one observational study that discuss the role of L-arginine in the treatment of MELAS, as shown in the PRISMA flow chart in Figure 1.

Study Characteristics and Outcomes

We included four studies that investigated the clinical efficacy of arginine in the treatment of MELAS. The characteristics of the studies included in this systematic review are presented in Table 2 [4,16-18]. The outcomes of the studies included in this systematic review are presented in Table 3 [4,16-18].

**Table 2:** Study characteristics and outcomes MELAS: mitochondrial encephalopathy, lactic acidosis, and stroke-like episodes; mtDNA: mitochondrial DNA.

Author and year of publication	Country	Study design	No. of patients in the treatment group	No. of patients in the control group	Patient selection	Dose, duration, and route of administration
Koga et al. (2018) [16]	Japan	Multicenter clinical trial	15 patients received oral L-arginine (13 finished the trial). 10 patients received intravenous L-arginine		Oral L-arginine clinical trial: 3243 A>G point mutation in mtDNA had not been treated with L-arginine previously. Patients had evaluable clinical manifestations of stroke-like episodes that presented in the past two years	IV (intravenous): L-arginine 0.5 g/kg per dose. Oral: L-arginine 0.3-0.5 g/kg/day divided into three doses after each meal for two years
Koga et al. (2006) [17]	Japan	Clinical trial	15	20	Genetic, pathologic, and clinical MELAS confirmation	Initially, one dose of IV L-arginine 0.5 g/kg of 10% solution. Then, oral L-arginine (0.15-0.3 g/kg/day) for 24 months
Koga et al. (2005) [18]	Japan	Clinical trial	24	72	Genetic, pathologic, and clinical MELAS confirmation	Acute phase: IV L-arginine infusion. Interictal phase: oral L-arginine (0.15-0.3 g/kg/day) for 18 months
Arakawa et al. (2010) [4]	Japan		6	6	Clinical and genetic MELAS confirmation	One dose of IV L-arginine (0.5 g/kg) within 30 minutes

**Table 3:** Study outcomes MELAS: mitochondrial encephalopathy, lactic acidosis, and stroke-like episodes; TCA: tricarboxylic acid; FMD: flow-mediated vasodilation; MBF: myocardial blood flow; PET: positron emission tomography; IV: intravenous, k_mono_: TCA-cycle metabolic rate.

Author, year	Outcome	Results	Conclusion
Koga et al. (2018) [16]	MELAS scale (oral L-arginine). Improvement of rates of headache, nausea, and vomiting after two hours post-IV L-arginine	Oral L-arginine decreased the severity and incidence of ictuses. Intravenous L-arginine decreased the frequency of nausea, vomiting, headache, visual disturbance, and impaired consciousness. Patients did not become bedridden, nor did the death occur during the trial or follow-up period. Ictuses were noted when L-arginine plasma concentration ≤ 167 µmol/L. No major side effects were reported	Oral L arginine offers clinical benefits, and IV L-arginine is well tolerated
Koga et al. (2006) [17]	Endothelial function (FMD) by measuring the brachial artery diameter response to hyperemic flow	After oral administration of L-arginine, FMD increased. MELAS patients have increased concentrations of amino acids than controls. Levels of L-arginine in MELAS patients before supplementation were inferior (36.4 ± 11.6 µmol/L) to controls (102.6 ± 15.7 µmol/L) during stroke-like episodes. After oral L-arginine supplementation, the frequency and severity of the stroke-like episodes decreased. During supplementation with L-arginine, patients with MELAS did not have a stroke-like attack. L-arginine supplementation improved FMD values after a period of two years of continuous administration (113 ± 2.4 µmol/L) compared to original values (104.7 ± 1.8 µmol/L) (p < 0.05) in MELAS patients. Post-two-hour IV administration of L-arginine showed increased FMD values (108.1 ± 2.6 µmol/L) compared to the original level (104.7 ± 2.6 µmol/L) (p < 0.05) in MELAS patients	L-arginine therapy improved endothelial dysfunction and showed promise in treating stroke-like episodes in MELAS
Koga et al. (2005) [18]	Severity and frequency of stroke-like episodes	Mean plasma concentrations of L-arginine and L-citrulline were lower in the interictal (84 ± 26 µmol/L and 26 ± 10 µmol/L) and acute phase (47 ± 13 µmol/L and 23± 10 µmol/L), respectively. The concentration of levels of L-arginine was significantly lower than in the interictal phase; meanwhile, levels of L-citrulline did not differ between the phases. Levels of nitric oxide were lower in the acute phase in MELAS patients (24 ± 10 µmol/L) (p < 0.01) versus control (45 ± 30 µmol/L) (p < 0.01), at the same time, in the interictal phase, nitric oxide levels were higher in patients with MELAS (91 ± 44 µmol/L) (p < 0.01), than in controls. The frequency of stroke-like episodes was 0.78 ± 0.42 before supplementation vs. 0.09 ± 0.09 after treatment (p < 0.05). The severity score before treatment was 2.04 ± 0.34 vs. after treatment was 0.17 ± 0.18 (p < 0.05)	L-arginine infusions significantly improved all stroke-like symptoms, suggesting that oral administration within 30 minutes of a stroke significantly decreased the frequency and severity of stroke-like episodes
Arakawa et al. (2010) [4]	Before and after administration of L-arginine: tricarboxylic acid (TCA) cycle metabolic rate (k_mono_). Dynamic C-11 acetate, PET imaging, myocardial efficiency, MBF	MELAS patients had a lower k_mono_ value than patients in the control group (0.051 ± 0.013 vs. 0.070 ± 0.019 min^-1^). Double product (DP)/k_mono _value was higher in MELAS patients than in the control group (1.69 ± 5.9 vs. 0.95 ± 1.2 x10^5^) and p < 0.005 vs. p < 0.004, respectively. No significant difference was shown between the distribution of k_mono_ and the increased MBF	The TCA cycle, which is suppressed in MELAS and switched to an anaerobic pathway, improves with the administration of L-arginine. Administration of L-arginine improves microcirculation, increases myocardial energy efficiency, and decreases myocardial oxidative metabolism

Study Limitations

Koga et al.’s study done in 2018 has several limitations, including a small population size, which reduces statistical impact. Also, it was not a placebo-controlled study due to ethical concerns leading to possible bias in selection, verification, and incorporation bias. Furthermore, the endpoints (improvement rates of nausea/vomiting and headache post two hours of L-arginine IV administration and MELAS stroke scale) were not reached [16].

Koga et al.’s study done in 2006 described no limitations [17]. While Koga et al.’s study in 2005 states that due to the studies being performed on the patients days or weeks after the onset of the stroke-like episode, secondarily induced nitric oxide (NO) production might alter the original pathophysiologic abnormality [18].

**Figure 2:**
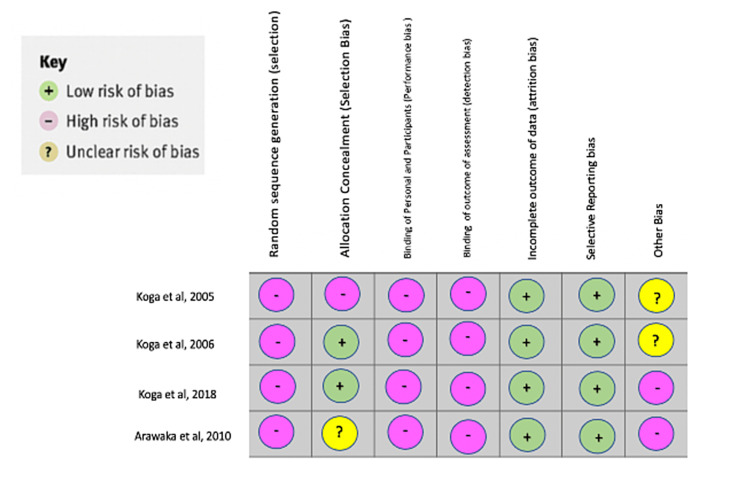
Bias analysis of this systematic review References [4,16-18].

In Arakawa et al.’s study, echocardiography was not performed immediately after L-arginine administration. Also, the patients with MELAS were much older and had fewer females compared to the control group. The statistical repercussion of this difference was not determined and should be analyzed in future studies [4]. Figure 2 shows the bias analysis of this systematic review [4,16-18].

Discussion

Synthesis of Citrulline, Arginine, and NO

There has been growing evidence that arginine and citrulline are deficient in MELAS. Clinical trials show evidence that patients with MELAS have lower levels of arginine and citrulline [17,19].

Arginine is synthesized from citrulline by two enzymes, argininosuccinate synthase and arginosuccinate [20]. Citrulline is synthesized mainly in the enterocytes by the following enzymes: pyrroline-5-carboxylate synthase (P5CS), ornithine aminotransferase (OAT), ornithine carbamoyltransferase (OCT), arginase II (ARG2), and proline oxidase (PO). MELAS is a mitochondrial disease, and because all these enzymes are found in the same organelle, it is only plausible to assume that there is a compromise of these enzymes in MELAS, which causes hypocitrullinemia in these patients [19]. As mentioned before, citrulline is the precursor of arginine, so a deficiency in citrulline will generate a deficiency in arginine as well [19].

**Figure 3:**
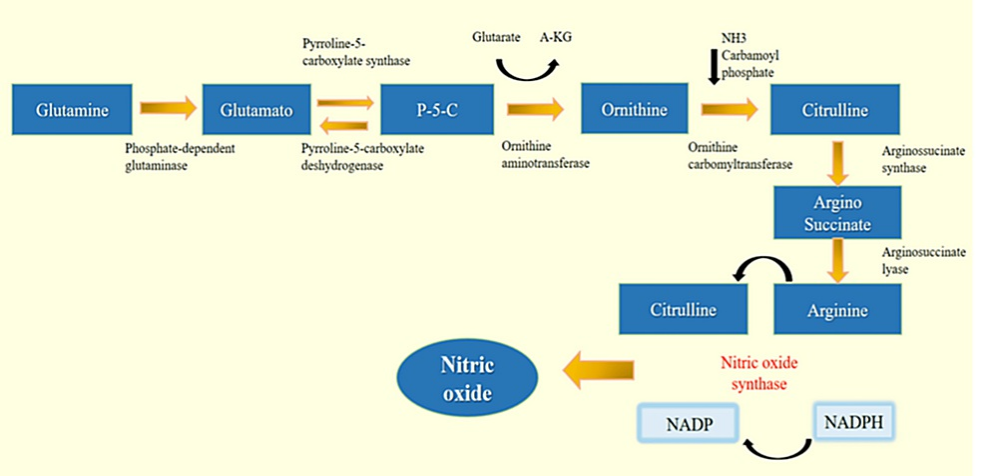
Process of synthesis of glutamine, citrulline, arginine, and NO NO: nitric oxide; P5C: pyrroline-5-carboxylic acid; NADP: nicotinamide adenine dinucleotide phosphate (oxidase form); NADPH: nicotinamide adenine dinucleotide phosphate (reduced form).

The lack of arginine and citrulline in MELAS patients generates a NO deficiency, which is synthesized from arginine and citrulline. Figure 3 shows the synthesis of glutamine, citrulline, arginine, and NO.

Role of Arginine and Citrulline in the Pathophysiology of MELAS

Arginine plasma levels were lower in the acute and interictal phases, while citrulline was only deficient in the acute phase, suggesting that arginine plays an important role in the pathophysiology of MELAS [18].

Regulation of the contractibility and tone of the vessels seems to be correlated with arginine levels. When there is a lack of arginine, NO cannot be produced in the endothelium. Deficient levels of arginine lead to vasoconstriction and decreased vascular remodeling [20].

Patients with MELAS also have an increased demand for NO production, which decreases even more arginine levels. There seems to be also increased clearance of arginine and citrulline in these patients. Finally, mitochondrial dysfunction could compromise the function of the enzymes that synthesize citrulline, especially the mitochondria of the enterocytes [21].

**Figure 4:**
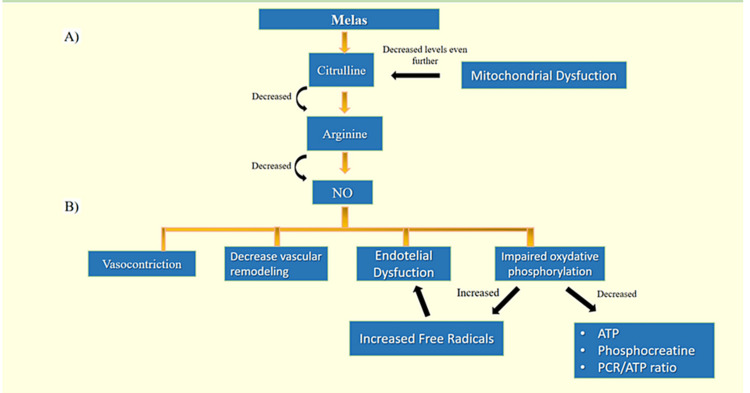
Pathophysiology of MELAS Part A: Pathophysiology of MELAS. Part B: Effects of decreased NO levels in MELAS. Low levels of arginine, citrulline, and NO lead to negative repercussions over the endothelial system. MELAS: mitochondrial encephalopathy, lactic acidosis, and stroke-like episodes; NO: nitric oxide; ATP: adenosine 5′-triphosphate; PCR: phosphocreatine.

Oxidative stress decreases levels of nitric oxide synthase, thereby decreasing, even more, the levels of NO in these patients [22]. Supplementation with arginine may restore the levels of NO in these patients restoring microcirculation. Oxidative stress caused by mitochondrial dysfunction possibly reduces NO production, while increasing dimethylarginine (a nitric oxide synthase inhibitor). Also, cytochrome c has excessive activity in MELAS patients. NO has a strong affinity to cytochrome c, contributing to the depletion of NO [21]. Finally, there is an increase in superoxide generation due to decreased levels of NO, worsening endothelial dysfunction [20]. Figure 4 shows the pathophysiology of MELAS.

Efficacy in Clinical Trials

Overall, these clinical trials have proposed the use of oral and IV arginine in the therapy of MELAS. IV arginine seems to be effective in improving symptoms during acute attacks of MELAS, while oral arginine supplementation increases endothelial function, preventing further stroke-like episodes.

The study by Koga et al. (2005) was the first publication to illustrate the importance of L-arginine in the endothelial vascular function in patients with MELAS during the acute and interictal phases [18]. This dysfunction, caused by insufficient nitric oxide-mediated vasodilation over the defective endothelium, can possibly be reversed with the administration of L-arginine [16]. Levels of L-arginine and L-citrulline in the MELAS group, compared to the placebo, were lower during the acute and interictal phases. Also, NO was found to be diminished in the acute phase [18]. Koga et al. (2018) later determined that plasma levels of arginine above 168 µmol/L may normalize endothelial dysfunction in MELAS patients [16].

In a clinical trial, Koga et al. (2006) found that L-arginine supplementation in patients with MELAS significantly improved endothelial function after normalizing plasma levels of this amino acid. Endothelial function measured by flow-mediated vasodilation (FMD) showed increased cerebral blood flow [17]. The distribution of cerebral blood flow was measured by statistical parametric mapping (SPM)-single photon emission computed tomography (SPECT). Furthermore, during L-arginine supplementation therapy, MELAS patients did not suffer any major stroke-like attacks, but they did present with headaches and teichopsia [17]. Patients with MELAS had lower plasma levels of L-arginine compared to controls. It was seen that after two years of supplementation with L-arginine, plasma levels improved from baseline reaching control levels. In addition, L-arginine improved endothelial function and prevented stroke-like episodes in MELAS patients. Oral administration of L-arginine reduced the severity and frequency of symptoms in MELAS patients and prevented further attacks [17].

Arakawa et al. (2010) explain that in MELAS patients, there is a failure in the respiratory chain that leads to a decrease in the tricarboxylic acid (TCA) cycle [4]. In this study, TCA was measured with k_mono_, which is an index of TCA activity. Suppressed TCA shifts energy production to the anaerobic pathway, causing lactic acidosis, which is characteristic of MELAS. The anaerobic metabolism paradoxically amplified myocardial efficiency compared to controls. Though the k_mono_ did not change significantly between MELAS and the control group overall, the patients with MELAS and treated with L-arginine were expected to have an increased k_mono_ index in relation to increased myocardial blood flow (MBF). However, there was no relation found between k_mono_ and MBF, suggesting that L-arginine enters and stimulates the TCA cycle. The study limitations, like disparities in age and sex, made the role of L-arginine administration unclear, calling for more studies to determine the role of this therapy in MELAS [4].

In the study of Koga et al. (2018), oral administration of L-arginine improved the MELAS stroke scale in these patients; at the same time, both oral and IV formulations were well tolerated [16]. Koga et al. describe a therapeutic regimen of L-arginine for MELAS during a nine-year, prospective, multicenter study. The research showed that L-arginine improved four predominant symptoms in MELAS: headache, nausea/vomiting, visual disturbances, and altered consciousness. In the two-year clinical trial and seven-year follow-up, there were no deaths nor were any patients bedridden. The study did not use a placebo in the control group due to strong ethical convictions, as it is a catastrophic disease. Having positive results, it recommends the strict use of L-arginine as a therapeutic measure to delay the course of this calamitous disease [16].

Future clinical trials with L-arginine

A non-randomized clinical trial executed in the Hospital for Sick Children in Canada aimed to study L-arginine therapy and its effects on stroke-like episodes was completed in 2013 [23]. The study was done with seven participants including three siblings (one male; two females) diagnosed with MELAS and four age and sex-matched controls. An interesting factor in this study is that the female participants with MELAS were matched with female controls that were on the same menstrual cycle. The menstrual cycle may play an important role when studying stroke-like episodes in MELAS patients since estrogen interferes with blood flow and arginine levels decrease during the luteal phase. This reduction may be attributed due to arginine’s immunomodulatory properties that are reduced to maintain viable pregnancy during the luteal phase [24,25]. The study used blood oxygen level-dependent (BOLD) functional MRI to observe changes in blood flow. Oral L-arginine was administered as a single dose and as a six-week trial, and its effects on blood vessel reactivity and brain cell activation, and aerobic function were analyzed. The results of this clinical trial demonstrated no difference in cerebral blood flow and cerebrovascular reactivity after six weeks of arginine supplementation [24]. The menstrual cycle stage variable considered in this study may serve as premises for further investigations on its influence on MELAS and its treatment.

Limitations

The studies analyzed in this systemic review are not open-label or randomized studies, and there is study heterogenicity among them. A double randomized clinical trial on this topic will be beneficial to further understand this disease and to establish an adequate treatment.

## Conclusions

Nitric oxide deficiency plays a potentially critical role in the pathophysiology of MELAS. The cause of this deficiency is multifactorial, probably due to a decreased synthesis of its precursors and/or an increased clearance, as seen in these patients; thereby suggesting a therapeutic approach by correcting this deficiency, which has shown promising results. According to the results of these studies, intravenous L-arginine is useful in the acute setting, while oral supplementation appears to increase endothelial function and prevent future stroke-like episodes. One of the proposed explanations for these results is that arginine, serving as a nitric oxide precursor, could potentially reverse the endothelial dysfunction seen in MELAS patients or at least ameliorate the symptomatology.
